# The Association of Previous Vaccination with Live-Attenuated Varicella Zoster Vaccine and COVID-19 Positivity: An Israeli Population-Based Study

**DOI:** 10.3390/vaccines10010074

**Published:** 2022-01-04

**Authors:** Eugene Merzon, Ilan Green, Eli Somekh, Shlomo Vinker, Avivit Golan-Cohen, Ariel Israel, Alessandro Gorohovski, Milana Frenkel-Morgenstern, Michal Stein

**Affiliations:** 1 Leumit Health Services, Tel Aviv 6473817, Israel; emarzon@leumit.co.il (E.M.); igreen@leumit.co.il (I.G.); svinker@leumit.co.il (S.V.); agolanchoen@leumit.co.il (A.G.-C.); aisrael@leumit.co.il (A.I.); 2 Adelson School of Medicine, Ariel University, Ariel 4076414, Israel; 3 Department of Family Medicine, Sackler Faculty of Medicine, Tel Aviv University, Tel Aviv 69978, Israel; 4 Department of Paediatrics, Mayanei Hayeshuah Medical Centre, Bnei Brak 5154475, Israel; esomeh@post.tau.ac.il; 5 Azrieli Faculty of Medicine, Bar-Ilan University, Safed 1311502, Israel; an.gorohovski@biu.ac.il; 6 Infectious Disease and Infection Control Unit, Hillel Yaffe Medical Center, Hadera 38100, Israel; MichalS@hy.health.gov.il; 7 Rappaport Faculty of Medicine, Technion, Israel Institute of Technology, Haifa 3525433, Israel

**Keywords:** COVID-19 infection, live-attenuated zoster vaccine

## Abstract

The Bacillus Calmette–Guérin (BCG) vaccine affords indirect protection against COVID-19, which is presumably due to priming of the innate immune system. It was hypothesized that the live attenuated Varicella Zoster (LAVZ) vaccine, recommended for the elderly population, would also protect against COVID-19 infection. A retrospective population-based cross-sectional study was conducted using the Leumit Health Services (LHS) database. LAVZ-vaccinated patients were matched with controls based on a propensity score model using 1:9 nearest-neighbor matching. Matching was based on age, gender, and the presence of some chronic disorders, which were selected according to their association with COVID-19 infection. Multivariate logistic regression analyses, adjusted for sex, age, smoking status, comorbidities, and chronic medications associated with COVID-19 risk, were used to estimate the association between LAVZ vaccination and COVID-19 RT-PCR results. Subjects (625) vaccinated with LAVZ and RT-PCR-tested for COVID-19 were identified. After 1:9 matching of subjects who received the LAVZ vaccine, 6250 subjects were included in the study. Multivariate logistic regression analysis demonstrated a significant and independent negative association between having received the LAVZ vaccine and the likelihood of COVID-19 infection (adjusted OR = 0.47 (95% CI 0.33–0.69, *p* < 0.001)). This association was further strengthened after separate analysis based on the time of LAVZ vaccination before COVID-19 RT-PCR testing. Individuals aged ≥50 years vaccinated with LAVZ had a decreased likelihood of being tested positive for COVID-19.

## 1. Introduction

Clinical observations have revealed that the beneficial effects of vaccines extend far beyond the direct effect on the disease for which they are intended. For instance, studies published in the last decade showed that children vaccinated against measles with the measles–mumps–rubella (MMR) vaccine showed a 30% reduction in mortality from any cause, although only 4% of the decline could be explained by the immunization against measles. Rather, the beneficial effect of the MMR vaccine appears to be derived in part from the direct heterologous benefits of measles vaccines that enhance innate and adaptive immune responses, as well as the prevention of measles and measles-associated short- and long-term immunomodulation effects [1,2]. Bacillus Calmette–Guérin (BCG) vaccine also provided a reduction in mortality far beyond reducing tuberculosis mortality in studies from Sweden, West Africa, and Guinea-Bissau [3,4,5]. The vaccine appeared to confer broad enhanced immunity to respiratory infections, even in elderly populations [3,4,6,7]. Moreover, several epidemiological studies have shown a beneficial effect of BCG vaccine on COVID-19 prognosis, with randomized clinical trials examining this association currently in progress [8,9,10]. Previous vaccination against influenza also provided a protective effect in terms of COVID-19 morbidity [11].

These indirect beneficial effects may be, at least in part, due to the innate immune system. The innate immune system is the first line of defense against invading pathogens. It is thought that priming or “training” of the innate immune system, which is defined as the enhancement of innate immune responses to subsequent infections, is achieved through epigenetic and metabolic programming of immune cells. This allows immune cells to mount a stronger response to pathogens and more efficiently activate adaptive responses [12,13,14,15,16,17].

COVID-19 morbidity is age-dependent, with older age having been documented as a major risk factor for increased morbidity and mortality from the disease [18,19,20]. As such, the administration of another vaccine recommended for the elderly, specifically, live attenuated Varicella Zoster (LAVZ) vaccine, may also influence innate immunity [21,22]. Accordingly, we hypothesized that the indirect stimulation of the immune system caused by the LAVZ vaccine may improve COVID-19 patient prognosis, much as with the BCG vaccine.

## 2. Methods

A retrospective population-based cross-sectional study was conducted utilizing data from the Leumit Health Services (LHS) database. LHS is a large nation-wide health maintenance organization (HMO) in Israel, which provides services to approximately 725,000 members. LHS maintains a comprehensive computerized database that is continuously updated in terms of member demographics, medical diagnoses, medical encounters, hospitalizations, and laboratory tests. All LHS members have similar health insurance and similar access to healthcare services. During each physician visit, a diagnosis is entered or updated according to the International Classification of Diseases, 9th revision (ICD-9) for somatic diagnoses and 10th revision (ICD-10) for psychiatric diagnoses. There is also an ongoing process of validation in which the physicians are encouraged to report on patients, who in their opinion, do not meet the criteria of certain diagnoses. As such, the validity of diagnoses entered into the registry is high for important medical diagnoses, in particular those based on laboratory data [23,24,25,26].

The study period was from 1 February to 30 November 2020 before Israel launched its COVID-19 vaccination campaign (20 December 2020). Testing for COVID-19 infection was performed as per physician referral, according to Israel Ministry of Health criteria for COVID-19 testing, which included direct exposure to a confirmed COVID-19 patient and/or presentation of symptoms suggesting COVID-19 (essentially, a cough, shortness of breath, or any other respiratory symptom, with fever). Nasopharyngeal swabs were taken and assessed for COVID-19 by real-time RT-PCR performed with internal positive and negative controls, according to World Health Organization guidelines [27].

The Allplex 2019-nCoV assay (Seegene, Seoul, Korea) was used until 10 March 2020, after which time the COBAS SARS-CoV-2 6800/8800 assay (Roche Pharmaceuticals, Basel, Switzerland) was employed. The study protocol was approved by the statutory clinical research committees of LHS and the Shamir Medical Center Institutional Review Board (Helsinki Committee approval # 0129-20-LEU, Shamir Medical Center, Be’er Yaakov, Israel) on human research.

### 2.1. Study Subjects

Data regarding demographics, laboratory results, and ICD-9 codes were derived from the LHS electronic medical records (EMR) system. COVID-19 RT-PCR testing of samples derived from nasopharyngeal swabs was performed by experienced personnel in a single centralized laboratory according to international guidelines [27]. All LHS enrollees who had been tested for COVID-19 during the study period were included in the present study. Subjects who purchased the LAVZ vaccine, according to pharmacy data, and received the injection, according to the specific nursing code, prior to COVID-19 RT-PCR testing were identified. Vaccinated patients were matched to controls using 1:9 ratios. Matching was based on age, gender, and the presence of certain chronic disorders (diabetes mellitus (DM), hypertension, ischemic heart disease (IHD), asthma and chronic obstructive pulmonary disease (COPD)), selected according to their association to COVID-19 infection [28].

### 2.2. Definitions

All diagnoses were based on ICD-9 codes for somatic disorders (such as DM, hypertension, IHD, malignancy, asthma, and COPD) and ICD-10 codes for psychiatric disorders (such as depression/anxiety and attention deficit hyperactivity disorder (ADHD)). Medication use was considered if the patient had been prescribed and purchased at least three prescriptions of certain groups of medications, which are defined according to their Anatomical Therapeutic Chemical (ATC) codes, namely, aspirin (acetylsalicylic acid; ATC code B01AC06), angiotensin-converting enzyme inhibitors (ACEIs; ATC code C09A and C09B), angiotensin II receptor blockers (ARBs; ATC- code C09C and C09D) and lipid-lowering medications (statins; ATC code C10A), during the past twelve months.

The LAVZ vaccine in Israel is offered to the population older than 50 years at a fee subsidized by supplementary insurance. Influenza vaccination in Israel is offered free of charge to all citizens older than 6 months, including HMO members; persons aged ≥65 years; and individuals with underlying chronic medical condition, who receive reminders before the influenza season.

The influenza vaccine is usually given during the fall, beginning in October, mainly in November, and occasionally through January and even February of the next year. In 2019–2020, the four-valent split influenza vaccines (Vaxigrip-Tetra, Sanofi-Pasteur, France or Fluarix-Tetra, GSK, Brentford, UK) were used; in 2018–2019, a three-valent vaccine (Influvac, Abbott, Chicago, IL, USA) was also used in <10% of vaccines. 

Obesity was defined as BMI > 30 kg/m^2^. Socioeconomic status (SES) data were organized according to the Israel Central Bureau of Statistics classification system that includes 20 sub-groups, delineated according to home address. Classifications one to nine are considered low–medium SES, while classifications 10–20 are considered upper medium–high SES. 

Smoking status was defined based upon the last EMR documentation recorded by the family physician. Rates of missing data were generally similar for varicella herpes-vaccinated subjects and controls. Levels of missing BMI and laboratory data were less than 10%.

### 2.3. Statistical Analysis

Differences in the demographic and clinical characteristics among subjects with negative and positive COVID-19 RT-PCR test results were analyzed using Student’s *t*-test and Fisher’s exact χ^2^ test for continuous and categorical variables, respectively, based on normal distribution and variable characteristics. Categorical data are presented as numbers and percentages. Data on continuous variables with normal distribution are presented as means and standard deviation (SD). We applied multiple imputations for missing data under the assumption that data were missing at random, based on the observed data. Preliminary evaluation of risk estimates (crude odds ratios) was conducted by stratified analyses. Subsequently, multivariate logistic regression analyses, adjusted for sex, age, smoking status, comorbidities, and chronic medications associated with COVID-19 risk [29,30,31,32] were used to estimate the adjusted odds ratio (OR) and 95% CI for the independent association between LAVZ vaccination and COVID-19 RT-PCR test results. The variance inflation factor (VIF) was used to check for multicollinearity among all variables in the model. All statistical analyses were conducted using the STATA 12 statistical package software (StataCorp, College Station, TX, USA). R functions (Shapiro–Wilk and Welch’s two-sample *t*-test) were used for statistical assessment of disease duration figures. Visualizations relied on open-source programs (Perl, R, GIMP, and Inkscape). The Ggplot2 R package (Hadley Wickham, Garrett Grolemund R for Data Science, O’Reilly Media, 2017) was used to produce figures, including scatter plots, area charts, bar charts, and 3D charts. Plots were prepared using in-house scripts written by the Frenkel–Morgenstern group in R and Perl.

## 3. Results

**Figure 1:**
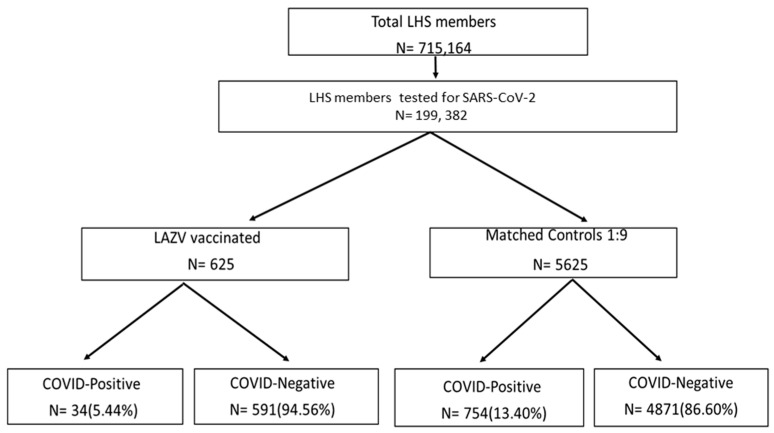
Flowchart of the study design.

A total of 625 subjects were identified who were vaccinated for Varicella Zoster (VZ) and tested by COVID-19 RT-PCR. After 1:9 matching of subjects who had not received the LAVZ vaccine, a total of 6250 subjects were included in the study (Figure 1). Comparisons of various demographic and clinical characteristics of vaccinated subjects and their controls are presented in Table 1. Rate of hospitalizations due to COVID-19 1 was significantly lower among LAVZ-vaccinated subjects as compared to non-vaccinated (1 (0.16%) vs. 21 (0.37%) *p* < 0.001). The COVID-19-positive group was younger (66.51 ± 12.7 years vs. 69.74 ± 12.34 years; *p* = 0.037), included a higher proportion of male gender (386 (48.9%) vs. 2332 (42.7%); *p* < 0.001), a higher proportion of persons from low-medium SES (506 (76.44%) vs. 5828 (59.38%); *p* < 0.001), and a significantly lower proportion of current smokers (60 (7.6%) vs. 685 (12.5%); *p* < 0.001) than the COVID-19-negative group.

The mean BMI was significantly higher for COVID-19-positive subjects (28.96 ± 5.32 vs. 28.12 ± 5.29, *p* < 0.001) (Table 2), as was the proportion of obese subjects (29 (35.15%) vs. 1611 (29.49%), *p* < 0.001) (Table 2). The prevalence of hypertension, ischemic heart disease asthma, COPD, and depression were significantly greater among persons who were COVID-19-negative (Table 2). In the COVID-19-positive group, greater prevalence of diabetes, malignancy, and ADHD were observed, although differences were not statistically significant (Table 2).

Multivariate logistic regression analysis, conducted after controlling for demographic variables and psychiatric and somatic disorders, demonstrated a significant and independent negative association between having been vaccinated with LAVZ vaccine and the likelihood of COVID-19 infection (adjusted OR = 0.47 (95% CI 0.33–0.69, *p* < 0.001)) (Table 3). Other significant and independent negative associations included having been vaccinated with seasonal influenza vaccine (adjusted OR = 0.83 (95% CI 0.69–0.99, *p* = 0.048)), a diagnosis of IHD (adjusted OR = 0.73 (95% CI 0.59–0.90, *p* < 0.001)), a diagnosis of asthma (adjusted OR = 0.59(95% CI 0.39–0.91, *p* < 0.001)), and having been treated with aspirin (OR = 0.78 (95% CI 0.63–0.97, *p* = 0.03)) and ARBs (OR = 0.65 (95% 0.47–0.90, *p* < 0.001)) (Table 3). Significant and independent positive associations included being a male (OR = 1.51 (95% CI 1.27)), residing in a low-medium SES city or town (OR = 2.58 (95% 2.16–3.09, *p* < 0.001)), being obese (adjusted OR = 1.27 (95% 1.06–1.52, *p* = 0.007)), and a diagnosis of DM (adjusted OR = 1.22 (95% CI 1.00–1.50, *p* = 0.043)) (Table 3). All VIFs were less than 3, which is indicative of a minor degree of multicollinearity in the data, which was not sufficient to warrant further assessment (Table 3).

**Figure 2:**
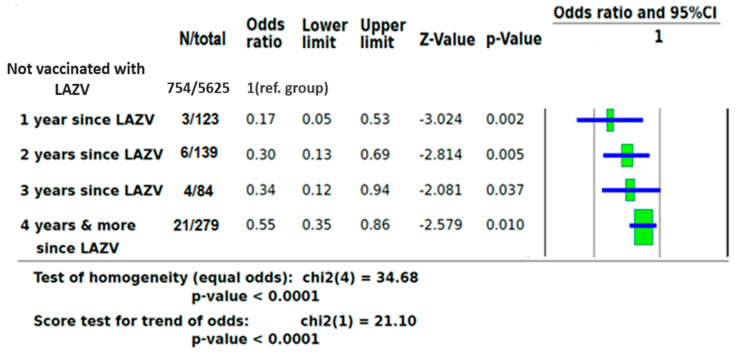
ORs of COVID-19 infection based on time of LAZV vaccination before PCR testing.

To strengthen the effect of LAVZ vaccine on the likelihood of COVID-19 PCR positivity, a separate analysis based on of time of vaccination before PCR testing was performed. As compared to individuals who were not vaccinated against VZ, those who were vaccinated one year before COVID-19 PCR testing had an OR of 0.17 (95% CI 0.05–0.53, *p* = 0.002), those vaccinated two years before COVID-19 PCR testing had an OR of 0.30 (95% CI 0.13–0.69, *p* = 0.005), those vaccinated three years before COVID-19 PCR testing had an OR of 0.34 (95% CI 0.12–0.94, *p* = 0.037), and those vaccinated four or more years before COVID-19 PCR testing had an OR of 0.55 (95% CI 0.35–0.86, *p* = 0.010) (Figure 2).

LAVZ vaccination was also associated with a decreased likelihood for hospitalization related to COVID-19 infection, but due to the small number of hospitalized subjects, this association was not statistically significant (crude OR of 0.43 (95% CI 0.06–3.18, *p* = 0.407)). In a multivariate analysis controlled for demographic variables and chronic disorders, the adjusted OR for hospitalization due to COVID-19 infection was 0.67 (95% CI 0.09–5.17, *p* = 0.701).

## 4. Discussion

This observational study reported a significant negative association between having been vaccinated with the LAVZ vaccine and the likelihood of a positive COVID-19 PCR test. Multivariate logistic regression demonstrated this association to be independent and not related to other variables, such as socioeconomic status, chronic administration of medications, and comorbidities, all of which were related to the susceptibility of COVID-19 infection in our previous studies [29,30,31,32]. A negative association between having received LAVZ vaccine and the likelihood of a positive COVID-19 PCR test was time-dependent: namely, the less time that had passed from the time of vaccination to the PCR test, the more pronounced was the negative association. This provides additional support for the assumption that the negative association between LAVZ vaccine and the likelihood of a positive COVID-19 PCR test is a real phenomenon and not a surrogate for the influences of unrelated confounders. Thus, these findings support previous evidence that prior stimulation of the immune system with vaccines provides indirect protection against COVID-19. In fact, the negative association with COVID-19 positivity as reported here was even superior to the negative association reported following vaccination with inactivated influenza vaccine described in our previous study [29]. At the same time, on the basis of epidemiological studies conducted in European countries, a protective effect of the BCG vaccine was postulated. Strong correlation was observed between the BCG index and COVID-19 mortality (*r*^2^ = 0.88; *p* = 8 × 10^−7^), indicating that every 10% increase in the BCG index was associated with a 10.4% reduction in COVID-19 mortality [8]. 

The proposed mechanism for this indirect effect is long-term imprinting (“training”) of innate immunity and myeloid cell function, which can be caused by microbial components, as well as by vaccines [14]. The cellular basis of trained immunity and heterologous protection against secondary infections resides in the functional reprogramming of innate immune cells by live attenuated vaccines (such as BCG) or microbial structures (such as beta-glucans and lipopolysaccharide). Such reprogramming can boost the anti-microbial functions of myeloid cells, as first demonstrated in invertebrates [20]. Further mouse model research [21] showed that the long-term induction of innate immune memory is generated through transcriptomic, epigenetic, epitopes similarity (Supplementary Material), and functional reprogramming of myeloid progenitors in the bone marrow. A recent study [22] of healthy volunteers demonstrated a myeloid-based bias in bone marrow hematopoietic stem cells three months after BCG vaccination.

This indirect protective mechanism, involving innate immune system induction and reprograming, may explain the significant minimal morbidity rate of COVID-19 seen in children. At a young age, the immune system is constantly being stimulated by vaccines; both live attenuated and adjuvanted, as well as by pathogens. With age, the frequency of vaccination decreases. At the same time, both the incidence rate and severity of COVID- 19 morbidity increase. Another possible explanation for the observed negative association between LAVZ vaccination and COVID-19 positivity could be molecular mimicry between COVID 19 and VZ vaccine antigens. Of course, further basic research is needed to confirm these theories.

## 5. Limitations

A major limitation of this observational and retrospective study is the lack of control over LAVZ vaccinations given to the study population. The LAVZ vaccine is offered to the elderly at a fee subsidized by supplementary insurance. Thus, it is likely that individuals who sought vaccination had a higher awareness of the importance of preventive care. To avoid this bias, we took sex, age, smoking status, socioeconomic status, chronic diseases and medications use, laboratory data, and comorbidities related to the risk of COVID-19 infection [29,30,31,32] into consideration by matching and applied adjustment to the multivariate analysis. However, unrecognized biases that influence our results could theoretically exist. Another serious limitation of this study is a lack of laboratory evidence that LAZV vaccine cause an indirect stimulation of the immune system.

The main strength of this study is that it is based on a population-based, comprehensive, solid, and reliable database.

In summary, this is the first description of the negative association between having been vaccinated with the LAVZ vaccine and the likelihood of COVID-19 positivity in adults ≥ 50 years. We believe that these results, if confirmed by subsequent studies, emphasize the importance of compliance with vaccination programs for direct and indirect protection.

## 6. Conclusions

We observed that individuals aged ≥ 50 years vaccinated with LAVZ had a decreased likelihood of testing positive for COVID-19. This epidemiological observation may support the theory of nonspecific stimulation of the innate immune system, and we try to explain why certain individuals are not infected with COVID-19. Of course, further epidemiological, as well as basic science, research are needed.

## Figures and Tables

**Table 1:** Demographic and clinical characteristics of LAZV-vaccinated subjects vs. non-vaccinated controls (1:9).

Demographics		LAZV-Vaccinated N = 625	Matched Control Group N = 5625	*p*-Value
Mean age (years ± SD)		69.35 ± 10.04	69.31 ± 13.15	0.751 *
Gender, N (%)	Male	272 (43.52%)	2446 (43.48%)	0.986 **
	Female	353 (56.48%)	3179 (56.52%)	
SES, N (%)	Low	179 (28.64%)	2603 (46.28%)	<0.001 **
	Middle–High	445 (71.2%)	2679 (47.63%)	
	Missing Data	1 (0.16%)	343 (6.1%)	
Smoking, N (%)	Never	535 (85.6%)	4093 (72.76%)	<0.001 **
	Current	57 (9.12%)	688 (12.23%)	
	Past	33 (5.28%)	844 (15%)	
BMI, Mean (SD)		27.77 ± 4.66	28.28 ± 5.37	0.022 *
Hemoglobin A1C, Mean (SD)		5.77 ± 0.85	5.79 ± 1.27	0.738 *
Obesity, N (%)		175 (28.18%)	1713 (30.45%)	0.124 **
Diabetes Mellitus, N (%)		271 (43.36%)	2465 (43.82%)	0.964 **
HTN, N (%)		366 (58.56%)	3292 (58.52%)	0.925 **
IHD, N (%)		179 (28.64%)	1598 (28.41%)	0.918 **
Asthma, N (%)		73 (11.68%)	654 (11.63%)	0.741 **
COPD, N (%)		107 (17.1%)	961 (17.08%)	0.643 **
Malignancy, N (%)		0	4 (0.07%)	0.504 **
Depression, N (%)		65 (10.40%)	673 (11.96%)	0.2502 **
ADHD, N (%)		18 (2.88%)	112 (1.99%)	0.1396 **
Aspirin, N (%)		224 (35.84%)	1803 (32.05%)	0.073 **
Statins, N (%)		370 (59.2%)	2251 (40.02%)	<0.001 **
ACEIs, N (%)		134 (21.44%)	1134 (20.16%)	0.450 **
ARBs, N (%)		94 (15.04%)	595 (10.58%)	<0.001 **
Flu vaccine (2019–2020), N (%)		486 (77.76%)	2544 (45.23%)	<0.001 **
Hospitalizations d/t COVID-19, N (%)		1 (0.16%)	21 (0.37%)	<0.001 **

ADHD—Attention Deficit Hyperactivity Disorder; CVD—Cardiovascular disease; COPD—Chronic Obstructive Pulmonary Disease; ACEIs—Angiotensin-converting enzyme inhibitors; ARBs—Angiotensin II receptor blockers, SES—Socioeconomic status; BMI—Body mass index, Obesity: BMI > 30. * Student’s *t*-test; ** Chi-squared test.

**Table 2:** Demographic and clinical characteristics of COVID-19-positive vs. COVID-19-negative subjects.

Demographics		COVID-19-Positive N = 788 (12.61%)	COVID-19-Negative N = 5462 (87.39%)	*p*-Value
Mean age (years ± SD)		66.50 ± 12.34	69.74 ± 12.73	<0.001 *
Gender, N (%)	Male	386 (48.98%)	2332 (42.69%)	<0.001 **
	Female	402 (51.02%)	3130 (57.31%)	
SES, N (%)	Low	524 (69.13%)	2258 (43.86%)	<0.001 **
	Middle-High	234 (30.87%)	2890 (56.14%)	
Smoking, N (%)	Never	609 (77.28%)	4019 (73.58%)	<0.001 **
	Current	60 (7.61%)	685 (12.54%)	
	Past	119 (15.10%)	758 (13.88%)	
BMI, Mean (SD)		28.96 ± 5.32	28.12 ± 5.29	<0.001 *
Hemoglobin A1C, Mean (SD)		5.85 ± 1.35	5.78 ± 1.22	0.192 *
Obesity, N (%)		277 (35.15%)	1611 (29.49%)	<0.001 **
Diabetes Mellitus, N (%)		348 (44.16%)	2388 (43.72%)	0.815 **
HTN, N (%)		414 (52.54%)	3244 (59.39%)	<0.001 **
IHD, N (%)		171 (21.70%)	1606 (29.40%)	<0.001 **
Asthma, N (%)		63 (7.99%)	664 (12.16%)	0.741 **
COPD, N (%)		113 (14.34%)	953 (17.45%)	0.0301 **
Malignancy, N (%)		1 (0.13%)	3 (0.05%)	<0.001 **
Depression, N (%)		74 (9.39%)	664 (12.16%)	0.024 **
ADHD, N (%)		21 (2.66%)	109 (2%)	0.218 **
Aspirin, N (%)		193 (24.49%)	1834 (33.58%)	<0.001 **
Statins, N (%)		260 (32.99%)	2359 (43.19%)	<0.001 **
ACEIs, N (%)		160 (20.30%)	1108 (20.29%)	0.990 **
ARSs, N (%)		57 (7.23%)	632 (11.57%)	<0.001 **
Flu vaccine (2019–2020), N (%)		304 (38.58%)	2726 (49.91%)	<0.001 **
LAZV vaccine, N (%)		34 (4.31%)	591 (10.82%)	<0.001 **

ADHD—Attention Deficit Hyperactivity Disorder; CVD—Cardiovascular disease; COPD—Chronic Obstructive Pulmonary Disease; ACEIs—Angiotensin-converting enzyme inhibitors; ARBs—Angiotensin II receptor blockers, SES—Socioeconomic status; BMI—Body mass index, Obesity: BMI > 30. * Student’s *t*-test; ** Chi-squared test.

**Table 3:** Multivariate analysis of variables associated with COVID-19 infection.

Variable	Crude OR	95% CI	*p*-Value	Adjusted * OR	95% CI	*p*-Value	VIF When All Covariates Are in the Model
Age	0.97	0.97–0.98	<0.001	0.99	0.98–1.00	0.130	1.03
Male gender	1.28	1.10–1.49	0.001	1.51	1.28–1.80	<0.001	1.76
Low SES	2.86	2.43–3.37	<0.001	2.58	2.16–3.09	<0.001	1.56
Smoking (current vs. never)	0.57	(0.40; 0.77)	<0.05	0.94	0.82–1.07	0.382	1.73
Diabetes Mellitus	1.01	0.87–1.18	0.815	1.22	1.00–1.50	0.043	1.45
Hemoglobin A1C	1.04	0.97–1.10	0.192	1.03	0.95–1.11	0.392	1.64
Obesity	1.29	1.10–1.51	0.002	1.27	1.06–1.52	0.007	1.89
HTN	0.74	0.64–0.86	<0.001	0.98	0.79–1.21	0.869	1.78
IHD	0.66	0.55–0.79	<0.001	0.73	0.59–0.90	0.005	2.01
Asthma	0.62	0.47–0.82	0.001	0.59	0.39–0.91	0.018	1.52
COPD	0.79	0.64–0.97	0.03	1.14	0.81–1.59	0.432	1.02
Malignancy	2.31	0.24–22.25	0.468	3.00	0.27–32.58	0.366	2.08
Depression	0.75	0.58–1.02	0.052	0.91	0.69–1.21	0.428	1.23
ADHD	1.34	0.84–2.16	0.220	1.31	0.76–2.22	0.337	1.11
Aspirin	0.64	0.54–0.76	<0.001	0.78	0.63–0.97	0.03	2.03
Statins	0.64	0.55–0.75	<0.001	0.85	0.70–1.04	0.134	2.56
ACEIs	1.00	0.83–1.20	0.990	1.02	0.81–1.28	0.833	1.34
ARBs	0.59	0.44–0.79	<0.001	0.65	0.47–0.90	<0.001	2.42
Flu vaccine (2019–2020)	0.63	0.54–0.73	<0.001	0.83	0.69–0.99	0.048	1.05
LAZV vaccine	0.37	0.26–0.52	<0.001	0.47	0.32–0.69	<0.001	1.94

ADHD—Attention Deficit Hyperactivity Disorder; CVD—Cardiovascular disease; COPD—Chronic Obstructive Pulmonary Disease; ACEIs—Angiotensin-converting enzyme inhibitors; ARBs—Angiotensin II receptor blockers, SES—Socioeconomic status; BMI—Body mass index, Obesity: BMI > 30. * Adjusted for age, gender, SES, smoking status, comorbidities, chronic medication use, and vaccinations.

## Data Availability

Data summary can be accessed after approval of research authorities of Leumit Health Services.

## Supplementary Materials

The following supporting information can be downloaded at: https://www.mdpi.com/article/10.3390/vaccines10010074/s1, Table S1: Spike-VZV protein identities: 5- and 6-mers.
