# Supplementary material for: The Association of Previous Vaccination with Live-Attenuated Varicella Zoster Vaccine and COVID-19 Positivity: An Israeli Population-Based Study
Source: Vaccines (Basel). 2022 Jan 4;10(1):74. doi: 10.3390/vaccines10010074 (PMC8777897; doi:10.3390/vaccines10010074)
Supplement: Supplementary file 1 [file vaccines-10-00074-s001.zip › vaccines-1387619-supplementary.pdf]

vaccines

The Varicella Zoster Virus fusion machinery

The varicella-zoster virus (VZV) contains a functional network comprising at least 5 proteins (gE, gI, gH, gL, and gB) directly involved in the adhesion processes, including virus-cell and virus-plasma membrane fusion, and cell-to-cell spread of the virus. The two proteins that make up the gE/gI heterodimer are thought to be responsible for the spread of VZV from cell to cell. A second heterodimer composed of the gH/gL proteins work together with gB to promote virus entry, due to its fusion to host membranes. In herpes virus, the conserved gB glycoprotein and the gH/gL heterodimer also mediate virion envelope fusion with cell membranes during virus entry. Moreover, it was shown that co-expression of VZV gB and gE, but not gB or gE alone, leads to abundant fusion and syncytium formation equivalent that seen upon gH and gL co-expression.

The VZV gB glycoprotein acts as a homotrimer, similarly to **the SARS-CoV-2 spike (S) glycoprotein**. Starting from the pre-fusion state, both proteins undergo significant conformational changes that allow them to adopt a post-fusion trimeric six-helix bundle fold that is used to trigger virus-cell and cell-cell fusion. We performed comparative analyses of the sequences of the VZV gB glycoprotein and SARS-CoV-2 S protein variants collected during the pandemic and found that due to on-going mutations, the S protein forms multiple 4,5, and 6-mer sequences identical to the proteins responsible for VZV fusion and spread. Specifically, these evolving sequence regions are in key regions significant for fusion dynamics, antibody recognition, and epitope presentation for the T- and B-cells in human population.

Results

For the most part, the VZV gB glycoprotein regions represented by 4-,5-, and 6-mers correspond to the S2′ domain of the spike glycoprotein (i.e., residues 816-1203, containing fusion peptides FP/FPPR and heptad repeats HR1/HR2). **Specifically, among 21 penta- and hexamers identified in our study, 11 pentamers and 2 hexamers were concentrated in the S2′ domain (Table 1).**

The C-terminal domain (CTD) of the S1 fragment, together with the FPPR (S2) sequence, are key components of the fusion machinery that modulates the fusogenic structural rearrangements of the S protein. When the receptor-binding domain (RBD) moves up, the FPPR sequence interacts with the CTD, thereby promoting the closed, RBD-down conformation of the pre-fusion spike protein trimer. The conformational dynamics of the spike protein was shown to accompany antibody binding to the RBD and proximal regions. **These data we are willing to incorporate in a new full-length manuscript**.

As such, our results show that the certain mutations in the spike protein variants create sequence patterns identical to those presented by VZV surface glycoproteins, including common B- and T-cell epitopes. Possible consequences of this phenomenon may include similarities in (a) immune responses to SARS-CoV-2 and VZV and (b) symptoms caused by both viruses. **These data we are willing to incorporate in a new full-length manuscript.**

Vaccines **2022**, *10*, 74. https://doi.org/10.3390/vaccines10010074 www.mdpi.com/journal/vaccines

Table S1. Spike-VZV protein identities: 5- and 6-mers

**VZV protein**	**5-mer**			**6-mer**			**Domain in the spike protein**
	**Seq**	**N1**	**N2**	**Seq**	**N1**	**N2**	
gE	RASVL	28	43				N-terminal domain (NTD)
	RQYGD	151	834				Fusion peptide proximal refion FPPR
	SGCTF	410	883				Fusion peptide proximal refion FPPR
	RLIEV	169	1181				Heptad repeat HR2
gI	IRTSA	97	1014				Central helix
gH	SYVTP	19	243				N-terminal domain (NTD)
	YLTTG	733	244				N-terminal domain (NTD)
	LNYIL	402	976				Heptad repeat HR1
gB	TSALL	900	873	TSALLT	900	873	Fusion peptide proximal region FPPR
	SALLT	901	874				Fusion peptide proximal region FPPR
	SQCVK	414	12				N-terminal domain (NTD)
	GDEIR	99	403				Receptor-binding domain (RBD)
	YLQEL	468	1198	YLQELV	468	1198	Heptad repeat HR2
	LQELV	469	1199				Heptad repeat HR2
	TPGTY	264	598				C-terminal domain 2 (CTD2)
	VEAFN	225	482				Receptor-binding motif (RBM)
	AGLVA	797	1221				Transmembrane domain (TM)
	LISIV	612	1221				Transmembrane domain (TM)
	ISETN	400	67				N-terminal domain (NTD)
